# [(*Z*)-*N*-(3-Chloro­phen­yl)-*O*-ethyl­thio­carbamato-κ*S*](triphenyl­phosphine-κ*P*)gold(I)

**DOI:** 10.1107/S1600536809049551

**Published:** 2009-11-25

**Authors:** Primjira P. Tadbuppa, Edward R. T. Tiekink

**Affiliations:** a Department of Chemistry, National University of Singapore, Singapore 117543; b Department of Chemistry, University of Malaya, 50603 Kuala Lumpur, Malaysia

## Abstract

The title compound, [Au(C_9_H_9_ClNOS)(C_18_H_15_P)], reveals a near linear geometry for the Au atom defined by a *S*,*P*-donor set [S—Au—P = 175.86 (3)°]. The deviation from linearity is ascribed to the proximate O atom derived from the thio­carbamato anion [Au⋯O = 2.967 (3) Å].

## Related literature

For structural systematics and luminescence properties of phosphinegold(I) carbonimidothio­ates, see: Ho *et al.* (2006 ▶); Ho & Tiekink (2007 ▶); Kuan *et al.* (2008 ▶). For the synthesis, see: Hall *et al.* (1993 ▶).
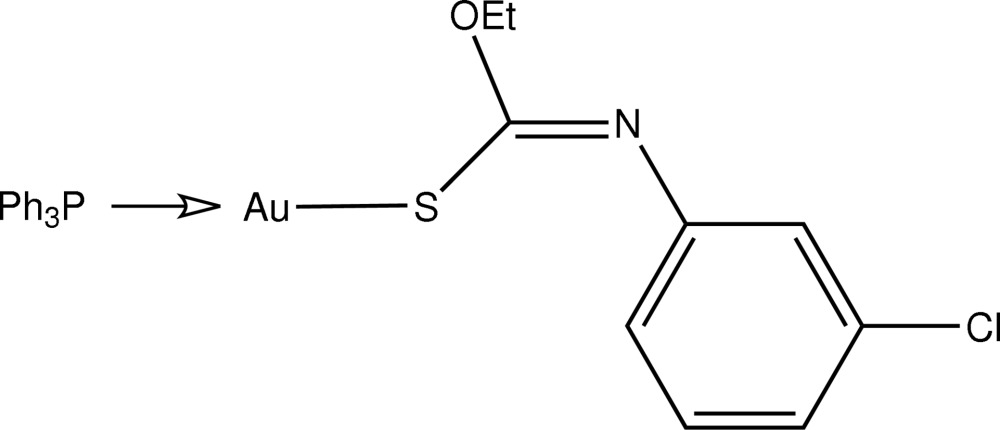


## Experimental

### 

#### Crystal data

[Au(C_9_H_9_ClNOS)(C_18_H_15_P)]
*M* *_r_* = 673.92
Triclinic, 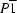

*a* = 8.7561 (4) Å
*b* = 12.3514 (6) Å
*c* = 13.0432 (6) Å
α = 110.076 (1)°
β = 105.289 (1)°
γ = 97.481 (1)°
*V* = 1239.52 (10) Å^3^
*Z* = 2
Mo *K*α radiation
μ = 6.21 mm^−1^
*T* = 223 K
0.11 × 0.10 × 0.05 mm

#### Data collection

Bruker SMART CCD area-detector diffractometer
Absorption correction: multi-scan (*SADABS*; Bruker, 2000 ▶) *T*_min_ = 0.620, *T*_max_ = 1
10396 measured reflections
5662 independent reflections
5184 reflections with *I* > 2σ(*I*)
*R*_int_ = 0.022

#### Refinement

*R*[*F* ^2^ > 2σ(*F* ^2^)] = 0.027
*wR*(*F* ^2^) = 0.065
*S* = 1.03
5662 reflections
298 parameters
H-atom parameters constrained
Δρ_max_ = 1.79 e Å^−3^
Δρ_min_ = −0.51 e Å^−3^

### 

Data collection: *SMART* (Bruker, 2000 ▶); cell refinement: *SAINT* (Bruker, 2000 ▶); data reduction: *SHELXTL* (Sheldrick, 2008 ▶); program(s) used to solve structure: *PATTY* in *DIRDIF92* (Beurskens *et al.*, 1992 ▶); program(s) used to refine structure: *SHELXL97* (Sheldrick, 2008 ▶); molecular graphics: *DIAMOND* (Brandenburg, 2006 ▶); software used to prepare material for publication: *publCIF* (Westrip, 2009 ▶).

## Supplementary Material

Crystal structure: contains datablocks global, I. DOI: 10.1107/S1600536809049551/ci2966sup1.cif

Structure factors: contains datablocks I. DOI: 10.1107/S1600536809049551/ci2966Isup2.hkl

Additional supplementary materials:  crystallographic information; 3D view; checkCIF report

## Figures and Tables

**Fig. 1.**
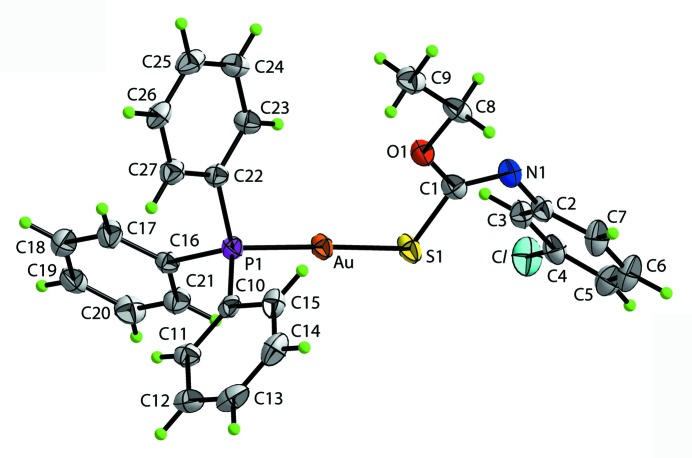
The molecular structure of the title compound, showing atom-labelling scheme and displacement ellipsoids at the 50% probability level.

## supplementary crystallographic information

### Comment

Systematic studies of phosphinegold(I) thiocarbamides (Ho *et al.* 2006; Ho & Tiekink, 2007; Kuan *et al.*, 2008), have been motivated by delineating crystal packing characteristics of these compounds, *e.g.* the propensity to form aurophilic (Au···Au) interactions, as well as examining their luminescence characteristics. The title compound, (C_5_H_5_)_3_PAu[SC(OEt)N(C_6_H_4_Cl-o)], was synthesized during the course of these studies.

The thiocarbamato anion functions as a thiolate ligand as seen in the magnitudes of the C1—S1 and C1═N1 bond distances of 1.759 (4) and 1.265 (4) Å, respectively; the conformation about the C1═N1 double bond is *Z*. The central SC(O)N chromophore is planar as seen in the S1–C1–N1–C2 and O1–C1–N1–C2 torsion angles of 2.0 (5) and -179.7 (3) °, respectively. The N-bound aryl ring is twisted out of this plane as seen in the C1–N1–C2–C3 torsion angle of 60.1 (5)°. The thiocarbamato and phosphine ligands define a *S*,*P* donor set. The deviation of the S1—Au—P1 angle [175.86 (3)°] from linearity is ascribed to the close approach of the O1 atom [2.967 (3) Å] to Au.

The crystal structure is dominated by π···π and C—H···π interactions. Centrosymmetrically related C16–C21 rings form π···π contacts: the *Cg*···*Cg*^i^ distance is 3.534 (2) Å; symmetry code (i) 1 - *x*, -*y*, -*z*. Two short C—H···π contacts are also noted, *viz*. C7—H7···*Cg*(C22—C27)^ii^ = 2.77 Å, C7···*Cg*(C22—C27)^ii^ = 3.630 (5) Å with an angle at H7 = 152 °; and C26—H26···*Cg*(C10—C15)^iii^ = 2.68 Å, C26···*Cg*(C10—C15)^iii^ = 3.560 (4) Å with an angle at H26 = 156 °; symmetry codes (ii) -*x*, 1 - *y*, -*z*; (iii) -*x*, -*y*, -1 - *z*.

### Experimental

The title compound was prepared following the standard literature procedure from the reaction of Ph_3_AuCl and EtOC(S)N(H)(C_6_H_4_Cl-*o*) in the presence of base (Hall *et al.*, 1993).

### Refinement

The H atoms were geometrically placed (C-H = 0.94–0.98 Å) and refined as riding with *U_iso_*(H) = 1.2–1.5*U*_eq_(C). A rotating group model was used for the methyl group. The maximum and minimum residual electron density peaks of 1.79 and 0.51 e Å^-3^, respectively, were located 0.85 Å and 1.44 Å from the Au atom.

### Crystal data

**Table:**

[Au(C_9_H_9_ClNOS)(C_18_H_15_P)]	*Z* = 2
*M**_r_* = 673.92	*F*(000) = 656
Triclinic, *P*1	*D*_x_ = 1.806 Mg m^−^^3^
Hall symbol: -P 1	Mo *K*α radiation, λ = 0.71069 Å
*a* = 8.7561 (4) Å	Cell parameters from 5349 reflections
*b* = 12.3514 (6) Å	θ = 2.5–29.7°
*c* = 13.0432 (6) Å	µ = 6.21 mm^−^^1^
α = 110.076 (1)°	*T* = 223 K
β = 105.289 (1)°	Block, colourless
γ = 97.481 (1)°	0.11 × 0.10 × 0.05 mm
*V* = 1239.52 (10) Å^3^	

### Data collection

**Table:**

Bruker SMART CCD area-detector diffractometer	5662 independent reflections
Radiation source: fine-focus sealed tube	5184 reflections with *I* > 2σ(*I*)
graphite	*R*_int_ = 0.022
ω scans	θ_max_ = 27.5°, θ_min_ = 1.8°
Absorption correction: multi-scan (*SADABS*; Bruker, 2000)	*h* = −11→11
*T*_min_ = 0.620, *T*_max_ = 1	*k* = −14→16
10396 measured reflections	*l* = −16→12

### Refinement

**Table:**

Refinement on *F*^2^	Primary atom site location: structure-invariant direct methods
Least-squares matrix: full	Secondary atom site location: difference Fourier map
*R*[*F*^2^ > 2σ(*F*^2^)] = 0.027	Hydrogen site location: inferred from neighbouring sites
*wR*(*F*^2^) = 0.065	H-atom parameters constrained
*S* = 1.03	*w* = 1/[σ^2^(*F*_o_^2^) + (0.0376*P*)^2^] where *P* = (*F*_o_^2^ + 2*F*_c_^2^)/3
5662 reflections	(Δ/σ)_max_ = 0.001
298 parameters	Δρ_max_ = 1.79 e Å^−^^3^
0 restraints	Δρ_min_ = −0.51 e Å^−^^3^

### Special details

**Table:**

Geometry. All s.u.'s (except the s.u. in the dihedral angle between two l.s. planes) are estimated using the full covariance matrix. The cell s.u.'s are taken into account individually in the estimation of s.u.'s in distances, angles and torsion angles; correlations between s.u.'s in cell parameters are only used when they are defined by crystal symmetry. An approximate (isotropic) treatment of cell s.u.'s is used for estimating s.u.'s involving l.s. planes.
Refinement. Refinement of *F*^2^ against ALL reflections. The weighted *R*-factor *wR* and goodness of fit *S* are based on *F*^2^, conventional *R*-factors *R* are based on *F*, with *F* set to zero for negative *F*^2^. The threshold expression of *F*^2^ > 2σ(*F*^2^) is used only for calculating *R*-factors(gt) *etc*. and is not relevant to the choice of reflections for refinement. *R*-factors based on *F*^2^ are statistically about twice as large as those based on *F*, and *R*- factors based on ALL data will be even larger.

### Fractional atomic coordinates and isotropic or equivalent isotropic displacement parameters (Å^2^)

**Table:**

	*x*	*y*	*z*	*U*_iso_*/*U*_eq_	
Au	0.129193 (15)	0.238137 (11)	0.027706 (10)	0.02816 (5)	
Cl	0.38116 (14)	0.55776 (11)	0.65290 (9)	0.0512 (3)	
S1	0.15605 (12)	0.37252 (8)	0.20892 (7)	0.0345 (2)	
P1	0.10947 (10)	0.09758 (8)	−0.14385 (7)	0.02494 (17)	
O1	0.2140 (3)	0.4991 (2)	0.0947 (2)	0.0326 (5)	
N1	0.1960 (4)	0.6110 (3)	0.2686 (3)	0.0331 (7)	
C1	0.1923 (4)	0.5102 (3)	0.1967 (3)	0.0303 (7)	
C2	0.1738 (4)	0.6210 (3)	0.3749 (3)	0.0312 (8)	
C3	0.2780 (4)	0.5892 (3)	0.4543 (3)	0.0336 (8)	
H3	0.3665	0.5596	0.4384	0.040*	
C4	0.2509 (4)	0.6011 (3)	0.5561 (3)	0.0338 (8)	
C5	0.1230 (5)	0.6436 (4)	0.5839 (3)	0.0409 (9)	
H5	0.1046	0.6490	0.6530	0.049*	
C6	0.0223 (5)	0.6783 (4)	0.5051 (4)	0.0459 (10)	
H6	−0.0647	0.7093	0.5222	0.055*	
C7	0.0478 (5)	0.6679 (4)	0.4030 (3)	0.0424 (9)	
H7	−0.0209	0.6929	0.3516	0.051*	
C8	0.2069 (5)	0.5990 (3)	0.0607 (3)	0.0362 (8)	
H8A	0.2921	0.6694	0.1178	0.043*	
H8B	0.1003	0.6180	0.0540	0.043*	
C9	0.2333 (5)	0.5609 (4)	−0.0537 (4)	0.0406 (9)	
H9A	0.2293	0.6245	−0.0812	0.061*	
H9B	0.1486	0.4908	−0.1089	0.061*	
H9C	0.3392	0.5426	−0.0454	0.061*	
C10	−0.0985 (4)	0.0188 (3)	−0.2372 (3)	0.0262 (7)	
C11	−0.1568 (4)	−0.1021 (3)	−0.2693 (3)	0.0308 (7)	
H11	−0.0861	−0.1468	−0.2463	0.037*	
C12	−0.3194 (4)	−0.1571 (4)	−0.3353 (3)	0.0379 (9)	
H12	−0.3587	−0.2389	−0.3564	0.045*	
C13	−0.4228 (4)	−0.0924 (4)	−0.3697 (3)	0.0416 (10)	
H13	−0.5328	−0.1299	−0.4142	0.050*	
C14	−0.3654 (5)	0.0279 (4)	−0.3389 (3)	0.0418 (9)	
H14	−0.4366	0.0718	−0.3630	0.050*	
C15	−0.2039 (4)	0.0843 (4)	−0.2728 (3)	0.0345 (8)	
H15	−0.1654	0.1661	−0.2522	0.041*	
C16	0.2160 (4)	−0.0128 (3)	−0.1206 (3)	0.0255 (7)	
C17	0.3041 (5)	−0.0661 (3)	−0.1893 (3)	0.0373 (8)	
H17	0.3025	−0.0508	−0.2553	0.045*	
C18	0.3940 (5)	−0.1415 (4)	−0.1609 (4)	0.0429 (9)	
H18	0.4555	−0.1759	−0.2068	0.051*	
C19	0.3949 (4)	−0.1669 (3)	−0.0666 (3)	0.0374 (8)	
H19	0.4568	−0.2183	−0.0478	0.045*	
C20	0.3047 (5)	−0.1168 (4)	0.0007 (4)	0.0413 (9)	
H20	0.3031	−0.1356	0.0645	0.050*	
C21	0.2165 (4)	−0.0390 (3)	−0.0253 (3)	0.0360 (8)	
H21	0.1568	−0.0038	0.0217	0.043*	
C22	0.1982 (4)	0.1559 (3)	−0.2303 (3)	0.0270 (7)	
C23	0.3065 (4)	0.2674 (3)	−0.1774 (3)	0.0306 (7)	
H23	0.3297	0.3125	−0.0982	0.037*	
C24	0.3804 (4)	0.3123 (3)	−0.2411 (3)	0.0354 (8)	
H24	0.4528	0.3879	−0.2053	0.042*	
C25	0.3477 (4)	0.2458 (4)	−0.3569 (3)	0.0369 (8)	
H25	0.3989	0.2761	−0.3997	0.044*	
C26	0.2395 (4)	0.1344 (4)	−0.4110 (3)	0.0341 (8)	
H26	0.2177	0.0896	−0.4901	0.041*	
C27	0.1640 (4)	0.0897 (3)	−0.3484 (3)	0.0320 (7)	
H27	0.0897	0.0148	−0.3851	0.038*	

### Atomic displacement parameters (Å^2^)

**Table:**

	*U*^11^	*U*^22^	*U*^33^	*U*^12^	*U*^13^	*U*^23^
Au	0.03676 (8)	0.02413 (8)	0.02236 (8)	0.00739 (5)	0.01005 (5)	0.00752 (5)
Cl	0.0597 (6)	0.0573 (7)	0.0413 (6)	0.0284 (5)	0.0125 (5)	0.0232 (5)
S1	0.0550 (5)	0.0237 (4)	0.0238 (4)	0.0093 (4)	0.0137 (4)	0.0079 (3)
P1	0.0299 (4)	0.0239 (4)	0.0207 (4)	0.0058 (3)	0.0096 (3)	0.0079 (3)
O1	0.0441 (14)	0.0262 (13)	0.0291 (13)	0.0099 (10)	0.0136 (10)	0.0109 (10)
N1	0.0367 (15)	0.0286 (16)	0.0304 (16)	0.0084 (12)	0.0098 (12)	0.0081 (12)
C1	0.0289 (16)	0.0289 (18)	0.0291 (17)	0.0059 (13)	0.0054 (13)	0.0103 (14)
C2	0.0325 (17)	0.0243 (17)	0.0290 (17)	0.0047 (13)	0.0094 (14)	0.0027 (14)
C3	0.0323 (17)	0.0282 (18)	0.0333 (19)	0.0090 (14)	0.0080 (14)	0.0054 (14)
C4	0.0368 (18)	0.0289 (18)	0.0277 (18)	0.0087 (14)	0.0043 (14)	0.0065 (14)
C5	0.044 (2)	0.047 (2)	0.032 (2)	0.0134 (18)	0.0151 (16)	0.0127 (17)
C6	0.040 (2)	0.058 (3)	0.040 (2)	0.0272 (19)	0.0174 (17)	0.013 (2)
C7	0.043 (2)	0.046 (2)	0.035 (2)	0.0221 (18)	0.0096 (16)	0.0107 (17)
C8	0.0394 (19)	0.0261 (18)	0.042 (2)	0.0057 (15)	0.0076 (16)	0.0172 (16)
C9	0.051 (2)	0.036 (2)	0.043 (2)	0.0110 (17)	0.0185 (18)	0.0225 (17)
C10	0.0280 (15)	0.0312 (18)	0.0196 (15)	0.0065 (13)	0.0126 (12)	0.0070 (13)
C11	0.0346 (17)	0.0335 (19)	0.0272 (17)	0.0055 (14)	0.0125 (14)	0.0147 (14)
C12	0.0345 (18)	0.043 (2)	0.0333 (19)	−0.0013 (16)	0.0128 (15)	0.0140 (16)
C13	0.0273 (17)	0.063 (3)	0.032 (2)	0.0043 (17)	0.0120 (15)	0.0154 (19)
C14	0.0369 (19)	0.058 (3)	0.038 (2)	0.0227 (18)	0.0159 (16)	0.0200 (19)
C15	0.0400 (19)	0.037 (2)	0.0305 (18)	0.0159 (16)	0.0162 (15)	0.0121 (15)
C16	0.0260 (15)	0.0230 (16)	0.0228 (15)	0.0022 (12)	0.0054 (12)	0.0068 (12)
C17	0.051 (2)	0.037 (2)	0.036 (2)	0.0180 (17)	0.0224 (17)	0.0182 (16)
C18	0.043 (2)	0.042 (2)	0.053 (2)	0.0162 (17)	0.0254 (18)	0.0208 (19)
C19	0.0345 (18)	0.0284 (19)	0.047 (2)	0.0068 (15)	0.0077 (16)	0.0161 (16)
C20	0.050 (2)	0.043 (2)	0.040 (2)	0.0135 (18)	0.0150 (17)	0.0259 (18)
C21	0.0414 (19)	0.038 (2)	0.037 (2)	0.0134 (16)	0.0184 (16)	0.0199 (16)
C22	0.0298 (16)	0.0279 (17)	0.0276 (16)	0.0088 (13)	0.0120 (13)	0.0135 (13)
C23	0.0298 (16)	0.0311 (18)	0.0280 (17)	0.0040 (14)	0.0067 (13)	0.0117 (14)
C24	0.0300 (17)	0.033 (2)	0.044 (2)	0.0038 (14)	0.0097 (15)	0.0198 (16)
C25	0.0353 (18)	0.049 (2)	0.042 (2)	0.0157 (16)	0.0198 (16)	0.0291 (18)
C26	0.0352 (18)	0.045 (2)	0.0271 (18)	0.0139 (16)	0.0155 (14)	0.0152 (16)
C27	0.0335 (17)	0.0342 (19)	0.0305 (18)	0.0086 (14)	0.0129 (14)	0.0135 (15)

### Geometric parameters (Å, °)

**Table:**

Au—P1	2.2588 (8)	C11—H11	0.94
Au—S1	2.3041 (9)	C12—C13	1.370 (6)
Cl—C4	1.745 (4)	C12—H12	0.94
S1—C1	1.759 (4)	C13—C14	1.383 (6)
P1—C22	1.807 (3)	C13—H13	0.94
P1—C16	1.813 (3)	C14—C15	1.384 (5)
P1—C10	1.817 (3)	C14—H14	0.94
O1—C1	1.356 (4)	C15—H15	0.94
O1—C8	1.451 (4)	C16—C17	1.386 (5)
N1—C1	1.265 (4)	C16—C21	1.387 (5)
N1—C2	1.416 (5)	C17—C18	1.378 (5)
C2—C7	1.386 (5)	C17—H17	0.94
C2—C3	1.392 (5)	C18—C19	1.368 (6)
C3—C4	1.372 (5)	C18—H18	0.94
C3—H3	0.94	C19—C20	1.377 (6)
C4—C5	1.377 (5)	C19—H19	0.94
C5—C6	1.392 (6)	C20—C21	1.384 (5)
C5—H5	0.94	C20—H20	0.94
C6—C7	1.375 (6)	C21—H21	0.94
C6—H6	0.94	C22—C23	1.391 (5)
C7—H7	0.94	C22—C27	1.400 (5)
C8—C9	1.494 (6)	C23—C24	1.387 (5)
C8—H8A	0.98	C23—H23	0.94
C8—H8B	0.98	C24—C25	1.377 (5)
C9—H9A	0.97	C24—H24	0.94
C9—H9B	0.97	C25—C26	1.389 (6)
C9—H9C	0.97	C25—H25	0.94
C10—C11	1.387 (5)	C26—C27	1.382 (5)
C10—C15	1.396 (5)	C26—H26	0.94
C11—C12	1.390 (5)	C27—H27	0.94
			
P1—Au—S1	175.86 (3)	C13—C12—C11	120.2 (4)
C1—S1—Au	103.15 (12)	C13—C12—H12	119.9
C22—P1—C16	106.60 (15)	C11—C12—H12	119.9
C22—P1—C10	104.86 (15)	C12—C13—C14	120.1 (4)
C16—P1—C10	107.11 (15)	C12—C13—H13	120.0
C22—P1—Au	113.34 (12)	C14—C13—H13	120.0
C16—P1—Au	110.08 (11)	C13—C14—C15	120.5 (4)
C10—P1—Au	114.33 (10)	C13—C14—H14	119.7
C1—O1—C8	117.8 (3)	C15—C14—H14	119.7
C1—N1—C2	119.6 (3)	C14—C15—C10	119.6 (4)
N1—C1—O1	120.3 (3)	C14—C15—H15	120.2
N1—C1—S1	127.7 (3)	C10—C15—H15	120.2
O1—C1—S1	111.9 (2)	C17—C16—C21	119.1 (3)
C7—C2—C3	118.6 (4)	C17—C16—P1	123.0 (3)
C7—C2—N1	119.2 (3)	C21—C16—P1	117.7 (3)
C3—C2—N1	122.2 (3)	C18—C17—C16	120.1 (4)
C4—C3—C2	119.6 (3)	C18—C17—H17	120.0
C4—C3—H3	120.2	C16—C17—H17	120.0
C2—C3—H3	120.2	C19—C18—C17	120.8 (4)
C3—C4—C5	122.6 (3)	C19—C18—H18	119.6
C3—C4—Cl	118.6 (3)	C17—C18—H18	119.6
C5—C4—Cl	118.7 (3)	C18—C19—C20	119.7 (4)
C4—C5—C6	117.2 (4)	C18—C19—H19	120.1
C4—C5—H5	121.4	C20—C19—H19	120.1
C6—C5—H5	121.4	C19—C20—C21	120.2 (4)
C7—C6—C5	121.2 (4)	C19—C20—H20	119.9
C7—C6—H6	119.4	C21—C20—H20	119.9
C5—C6—H6	119.4	C20—C21—C16	120.1 (3)
C6—C7—C2	120.7 (4)	C20—C21—H21	119.9
C6—C7—H7	119.6	C16—C21—H21	119.9
C2—C7—H7	119.6	C23—C22—C27	119.4 (3)
O1—C8—C9	105.7 (3)	C23—C22—P1	119.2 (3)
O1—C8—H8A	110.6	C27—C22—P1	121.4 (3)
C9—C8—H8A	110.6	C24—C23—C22	120.2 (3)
O1—C8—H8B	110.6	C24—C23—H23	119.9
C9—C8—H8B	110.6	C22—C23—H23	119.9
H8A—C8—H8B	108.7	C25—C24—C23	120.0 (3)
C8—C9—H9A	109.5	C25—C24—H24	120.0
C8—C9—H9B	109.5	C23—C24—H24	120.0
H9A—C9—H9B	109.5	C24—C25—C26	120.5 (3)
C8—C9—H9C	109.5	C24—C25—H25	119.8
H9A—C9—H9C	109.5	C26—C25—H25	119.8
H9B—C9—H9C	109.5	C27—C26—C25	119.9 (3)
C11—C10—C15	119.5 (3)	C27—C26—H26	120.0
C11—C10—P1	122.3 (3)	C25—C26—H26	120.0
C15—C10—P1	118.2 (3)	C26—C27—C22	120.0 (3)
C10—C11—C12	120.2 (4)	C26—C27—H27	120.0
C10—C11—H11	119.9	C22—C27—H27	120.0
C12—C11—H11	119.9		
			
C2—N1—C1—O1	−179.7 (3)	C11—C10—C15—C14	0.6 (5)
C2—N1—C1—S1	2.0 (5)	P1—C10—C15—C14	−176.6 (3)
C8—O1—C1—N1	−12.7 (5)	C22—P1—C16—C17	−19.4 (3)
C8—O1—C1—S1	165.9 (2)	C10—P1—C16—C17	92.4 (3)
Au—S1—C1—N1	170.7 (3)	Au—P1—C16—C17	−142.7 (3)
Au—S1—C1—O1	−7.7 (2)	C22—P1—C16—C21	156.6 (3)
C1—N1—C2—C7	−122.2 (4)	C10—P1—C16—C21	−91.6 (3)
C1—N1—C2—C3	60.1 (5)	Au—P1—C16—C21	33.3 (3)
C7—C2—C3—C4	2.1 (5)	C21—C16—C17—C18	−1.7 (5)
N1—C2—C3—C4	179.8 (3)	P1—C16—C17—C18	174.3 (3)
C2—C3—C4—C5	0.2 (6)	C16—C17—C18—C19	1.4 (6)
C2—C3—C4—Cl	178.8 (3)	C17—C18—C19—C20	0.1 (6)
C3—C4—C5—C6	−1.9 (6)	C18—C19—C20—C21	−1.5 (6)
Cl—C4—C5—C6	179.5 (3)	C19—C20—C21—C16	1.3 (6)
C4—C5—C6—C7	1.4 (7)	C17—C16—C21—C20	0.3 (5)
C5—C6—C7—C2	0.8 (7)	P1—C16—C21—C20	−175.8 (3)
C3—C2—C7—C6	−2.6 (6)	C16—P1—C22—C23	−103.6 (3)
N1—C2—C7—C6	179.6 (4)	C10—P1—C22—C23	143.0 (3)
C1—O1—C8—C9	−179.6 (3)	Au—P1—C22—C23	17.6 (3)
C22—P1—C10—C11	119.2 (3)	C16—P1—C22—C27	74.4 (3)
C16—P1—C10—C11	6.1 (3)	C10—P1—C22—C27	−39.0 (3)
Au—P1—C10—C11	−116.1 (3)	Au—P1—C22—C27	−164.4 (2)
C22—P1—C10—C15	−63.8 (3)	C27—C22—C23—C24	−0.3 (5)
C16—P1—C10—C15	−176.8 (3)	P1—C22—C23—C24	177.8 (3)
Au—P1—C10—C15	61.0 (3)	C22—C23—C24—C25	−0.6 (5)
C15—C10—C11—C12	−0.8 (5)	C23—C24—C25—C26	0.7 (6)
P1—C10—C11—C12	176.3 (3)	C24—C25—C26—C27	0.0 (6)
C10—C11—C12—C13	0.4 (5)	C25—C26—C27—C22	−0.8 (5)
C11—C12—C13—C14	0.1 (6)	C23—C22—C27—C26	1.0 (5)
C12—C13—C14—C15	−0.3 (6)	P1—C22—C27—C26	−177.0 (3)
C13—C14—C15—C10	0.0 (6)		
